# Data Mining of Determinants of Intrauterine Growth Retardation Revisited Using Novel Algorithms Generating Semantic Maps and Prototypical Discriminating Variable Profiles

**DOI:** 10.1371/journal.pone.0126020

**Published:** 2015-07-09

**Authors:** Massimo Buscema, Enzo Grossi, Luisa Montanini, Maria E. Street

**Affiliations:** 1 Semeion Research Centre of Sciences of Communication, Rome, Italy; 2 Centre for Mathematical and Computational Biology, Dept. of Mathematical and Statistical Sciences, University of Colorado at Denver, Denver, CO, United States of America; 3 Villa Santa Maria Institute, Tavernerio, Italy; 4 Department of Pediatrics, University Hospital of Parma, Parma, Italy; 5 Department of Pediatrics, IRCCS- Arcispedale S. Maria Nuova, Reggio Emilia, Italy; Medical Faculty, Otto-von-Guericke University Magdeburg, Medical Faculty, GERMANY

## Abstract

**Objectives:**

Intra-uterine growth retardation is often of unknown origin, and is of great interest as a “Fetal Origin of Adult Disease” has been now well recognized. We built a benchmark based upon a previously analysed data set related to Intrauterine Growth Retardation with 46 subjects described by 14 variables, related with the insulin-like growth factor system and pro-inflammatory cytokines, namely interleukin -6 and tumor necrosis factor -α.

**Design and Methods:**

We used new algorithms for optimal information sorting based on the combination of two neural network algorithms: Auto-contractive Map and Activation and Competition System. Auto-Contractive Map spatializes the relationships among variables or records by constructing a suitable embedding space where ‘closeness’ among variables or records reflects accurately their associations. The Activation and Competition System algorithm instead works as a dynamic non linear associative memory on the weight matrices of other algorithms, and is able to produce a prototypical variable profile of a given target.

**Results:**

Classical statistical analysis, proved to be unable to distinguish intrauterine growth retardation from appropriate-for-gestational age (AGA) subjects due to the high non-linearity of underlying functions. Auto-contractive map succeeded in clustering and differentiating completely the conditions under study, while Activation and Competition System allowed to develop the profile of variables which discriminated the two conditions under study better than any other previous form of attempt. In particular, Activation and Competition System showed that ppropriateness for gestational age was explained by IGF-2 relative gene expression, and by IGFBP-2 and TNF-α placental contents. IUGR instead was explained by IGF-I, IGFBP-1, IGFBP-2 and IL-6 gene expression in placenta.

**Conclusion:**

This further analysis provided further insight into the placental key-players of fetal growth within the insulin-like growth factor and cytokine systems. Our previous published analysis could identify only which variables were predictive of fetal growth in general, and identified only some relationships.

## Introduction

Most of the data concerning determinants of fetal growth restriction or intrauterine growth retardation (IUGR) come from traditional statistical analysis, which is unable to grasp complex interactions among variables when the underlying functions are non linear. Many IUGR cases are still of unknown origin [1]. The interest in IUGR has grown because approximately 13% of these subjects do not present a catch-up growth [2], and in recent years, the concept of a “Fetal Origin of Adult Disease” has been introduced to describe modifications in utero that can influence adult life [3].

In a previous paper [4], we showed that using supervised Artificial Neural Networks (ANN) it was possible to predict the presence or absence of IUGR with a high degree of accuracy starting from biomarkers of uterine patho-physiology belonging to the insulin-like growth factor (IGF) system, and Interleukin (IL)-6.

The IGF system consists in two main peptides, IGF-I and IGF-2, and in six main binding proteins, the IGF binding proteins (IGFBP) which regulate their biological activity. The IGF system is recognized to be crucial for fetal growth, as experiments in knockout mice have shown [5–8]. It is well known that IGF-I and IGF-2 are both synthesized in the placenta [9–11]. IGFBP-1, IGFBP-2, IGFBP-4 and IGFBP-6 are also expressed by all placenta cell types while IGFBP-3 and IGFBP-5 are expressed only by some [12].

Pro-inflammatory cytokines are recognized to be important for placental growth and development, however, not much research is available today in particular in relationship with idiopathic intrauterine growth retardation [4,13–15].

In our previous paper the use of a first release connectivity map showed that IGF-2 concentrations in placental lysates was connected with its gene expression, with mother’s age at delivery, and with IL-6 and IGFBP-2 placental contents, and that appropriateness for gestational age was related with gestational age but not clearly with any of the determinants identified within the IGF and cytokine systems [4]. In a following study, using Bayesian networks for the IUGR subjects we could identify a clear role for IL-6, and IGF-2 that seemed to act by the intermediation of IL-6. A direct relationship with IGFBP-2 and TNF-α placental contents was identified also [16].

In order to improve and better address the problem of data mining in complex systems like this one under study, we addressed the problem with a novel kind of algorithms able to identify hidden relationships between variables, to cluster properly when applied to the records, and to generate prototypical variable profiles with the aim to discriminate between normal and abnormal fetal growth.

The ultimate aim of a similar analysis in medicine is to underpin possible therapeutic targets, and obtain a better and more complete understanding of a systems biology compared with traditional approaches.

This study had two main aims: i) understand the differences between normal and abnormal fetal growth providing a study of the system’s biology in the two different conditions (appropriate for gestational age-AGA and IUGR); ii) identify hidden relationships between variables related to intrauterine growth retardation and generate prototypical variable profiles, i.e. perform data mining to provide a better understanding of the changes that occur in a given condition.

## Materials and Methods

### Subjects

Twenty IUGR and 26 AGA pregnancies were included in the study as previously reported [4,13,16]. All pregnancies were dated correctly by ultrasound during the first trimester of gestation. All neonates, both IUGR and AGA, were delivered by elective caesarean section (CS). Cases with increased blood pressure, gestational diabetes, or reduced amniotic fluid were not included in the study as previously stated [13].

As previously, described, AGA births were defined on the basis of a normal birth weight (<80th and >10th centile) with respect to the Italian standards of referral [17], a normal pregnancy and the absence of maternal risk factors [13].

The IUGR pregnancies were defined and diagnosed by ultrasound according to the following criteria: abdominal circumference <10th centile and shift of fetal growth with a reduction of abdominal circumference with respect to the measure taken within the 20th week of gestation. The diagnosis was made within the 32nd week of gestation and was ascribed to a probable placental cause after excluding infections, chromosomal abnormalities, genetic syndromes, maternal malnutrition, substance abuse, gross placental abnormalities and multiple fetuses [13].

### Variables

At birth the following information was collected: maternal age, weight at birth of both parents, body mass index (BMI) of the mother before pregnancy, previous gynecological history, medical history during pregnancy, fetal biophysical data (exact duration of pregnancy, growth trend, fetal and maternal doppler velocimetry data in IUGR, Non Stress Test), clinical data at delivery (indication for CS, neonatal sex, weight, length, head circumference, Apgar score, acid-base equilibrium, and perinatal data), and weight and macroscopic appearance of the placenta [13].

### Ethical Approval

Written informed consent was obtained from the mothers as appropriate. The study was approved by the local Ethics Committee (University of Parma Medical School).

### Collection of Biological Material

In all cases, four fragments of perifunicular villous tissue of approximately 5 mm^3^ were taken close to the fetal plate, rinsed repeatedly in sterile saline solution at 0°C. Storage conditions were standardized as previously described [13].

### Isolation of RNA

RNA extraction was performed as previously described [13].

### cDNA Synthesis

Complementary DNA (cDNA) was synthesized using 1μg of total RNA sample, previously treated with DNAse, according to the recommendations of the manufacturer (Applied Biosystems, Foster City, California), and as previously described [13].

### TaqMan Assay on Demand Gene Expression

Real-Time Quantitative RT-PCR was performed on a TaqMan ABI 7700 Sequence Detector System (Applied Biosystems) as previously described [13,18]. Applied Biosystems TaqMan Assay-on-Demand Gene Expression pre-designed primers and probes were used.

### Total Protein Content

The lysates were extracted as previously described [13]. The total protein content was expressed in μg per mg of total protein content in the placenta.

### Protein Assays

Total IGF-I, IGF-2, IGFBP-2 and IL-6 were measured as previously described [13]. TNF-α was assayed using an ultrasensitive ELISA method ((Biosource International Camarillo, CA, USA). The sensitivity of the method was < 0.09 pg/ml, the intra- and inter-assay coefficients of variation were 6.7 and 7.7%, respectively. All concentrations were normalized per mg of total placental protein content.

### Database and Data Analysis

Artificial Neural Networks (ANNs) analysis cannot be performed on incomplete data. We aimed to re-analyze from a completely new perspective most of the data we obtained from our previous study, comparing IUGR and AGA newborns [4,13,16].

Fourteen variables were selected for the analysis from the entire database of 46 subjects: IUGR and AGA membership, gender, gestational age, total placental protein content per mg of placental tissue (PRO μg/mg), relative gene expression for IGF-I, IGF-2, IGFBP-1, IGFBP-2, and IL-6, (abbreviated as mRNA_IGF1, mRNA_IGF2, mRNA-BP1, mRNA_BP2, mRNA_IL6, respectively), and placental lysate content in IGF-2, IGFBP-2, TNF-α, and IL-6 (abbreviated as: PLA_IGF2, PLA_BP2, PLATNF, and PLAIL6 respectively).

### Basic Statistics

The linear correlation index between variables was calculated. Simple Student’s-T test was used to compare R squared between each variable and IUGR and AGA targets in the two groups of variables.

### Classic Algorithms

Different algorithms were applied to the dataset and its results compared with the real class to which each subject belonged: i) K-mean clustering was performed according the method described by Rousseeuw [19] (in short K-Mean); ii) Minimum Spanning Tree (MST) Clustering based on Linear Correlation (in short LC MST); iii) Principal Component Analysis (PCA) was applied on the dataset (implementation from MatLab ToolBox) and then its two main components were post-processed with the Minimum Spanning Tree (in short PCA MST); iv) Linear Discriminant Analysis (LDA) based on the input generated by PCA (in short PCA-LDA); v) Self Organizing Maps (SOM) with a matrix 10x10 run for 100 epochs (software implementation by Matlab ToolBox) and filtered by MST (in short SOM MST); vi) LDA based on SOM codebooks (in short SOM LDA).

As LDA is a supervised algorithm we used the Leave One Out protocol to evaluate the results. In this way we applied the algorithm on the whole sample.

### Artificial Neural Networks Analysis

We subsequently used new and powerful ANNs: i) (Auto Contractive Map) AutoCM, a new non-linear ANN designed in 1999 by M. Buscema at the Semeion Research Center. AutoCM algorithm was previously applied in medicine with very interesting results [20–23]; ii) (Activation and Competition System) ACS, a new non-linear Auto Associative Memory, created by M. Buscema at the Semeion Research Center [24].

The theories and mathematical details of the two ANNs are described in detail below.

### AutoCM Artificial Neural Network

AutoCM ‘spatializes’ the correlation among variables by building a suitable embedding space where a visually transparent and cognitively natural notion such as ‘closeness’ among variables reflects accurately their associations. AutoCM converts this ‘closeness’ into a compelling graph-theoretical representation that picks all and only the relevant correlations and organizes them into a coherent picture. Such representation is not actually built through a cumbersome aggregation of two-by-two associations between couples of variables, but rather by building a complex global picture of the whole pattern of variation. Moreover, it fully exploits the topological meaning of graph-theoretical representations in that actual paths connecting vertices (variables) in the representation carry a definite meaning in terms of logical interdependence in explaining the data set’s variability.

The AutoCM is characterized by a three-layer architecture: an Input layer, where the signal is captured from the environment, a Hidden layer, where the signal is modulated inside the AutoCM, and an Output layer, through which the AutoCM feeds back upon the environment on the basis of the stimuli previously received and processed.

Each layer contains an equal number of N units, so that the whole AutoCM is made of 3N units. The connections between the Input and the Hidden layers are mono-dedicated, whereas, the ones between the Hidden and the Output layers are fully saturated, i.e. at maximum gradient. Therefore, given N units, the total number of the connections, Nc, is given by: *Nc = N (N + 1)*.

All of the connections of AutoCM may be initialized either by assigning a same, constant value to each, or by assigning values at random. The best practice is to initialize all the connections with a same, positive value, close to zero.

The learning algorithm of AutoCM may be summarized in a sequence of four characteristic steps: i) Signal Transfer from the Input into the Hidden layer; ii) Adaptation of the values of the connections between the Input and the Hidden layers; iii) Signal Transfer from the Hidden into the Output layer; iv) Adaptation of the value of the connections between the Hidden and the Output layers.

Notice that steps ii and iii may take place in parallel.

m[s] are the units of the Input layer (sensors), scaled between 0 and 1; m[h] the units of the Hidden layer, and m[t] the units of the Output layer (system target). Moreover, the vector of mono-dedicated connections is defined v; the matrix of the connections between the Hidden and the Output layers as w; p is the index for each pattern and M the global number of patterns; and the discrete time that spans the evolution of the AutoCM weights, or, put in another way, the number of epochs of processing, (one epoch is completed when all the patterns are inputted) is n: *n*∈*T*.

In order to specify the steps i-iv that define the AutoCM algorithm, we defined the corresponding signal forward-transfer equations and the learning equations, as follows:

- a. Signal transfer from the Input to the Hidden layer:

$$m_{i,p_{\left(n\right)}}^{\left[h\right]}=m_{i,p}^{\left[s\right]}\cdot \left(1-\frac{v_{i_{\left(n\right)}}^{}}{C}\right);$$ (1)

where *C* is a positive real number not lower than 1, which we will refer to as the contraction parameter (see below for comments), and where the (*n*) subscript has been omitted from the notation of the input layer units, as these remain constant at every cycle of processing. It is useful to set $C=\sqrt[{2}]{N}$, where N is the number of variables considered. The Learning Coefficient, α, is set as $\alpha =\frac{1}{M};$

- b. Adaptation of the connections $v_{i_{\left(n\right)}}$ through the variation $\Delta v_{i_{\left(n\right)}}$, which amounts to trapping the energy difference generated according to Eq (1):

$$\Delta v_{i_{\left(n\right)}}=\sum_{p}^{M}\left(m_{i,p}^{\left[s\right]}-m_{i,p_{\left(n\right)}}^{\left[h\right]}\right)\cdot \left(1-\frac{v_{i_{\left(n\right)}}^{}}{C}\right)\cdot m_{i,p}^{\left[s\right]};$$ (2)

$$v_{i_{\left(n+1\right)}}=v_{i_{\left(n\right)}}+\alpha \cdot \Delta v_{i_{\left(n\right)}}$$ (3)

- c. Signal transfer from the Hidden to the Output layer:

$$Net_{i,p_{\left(n\right)}}=\sum_{j=1}^{N}m_{j,p_{\left(n\right)}}^{\left[h\right]}\cdot \left(1-\frac{w_{i,j_{\left(n\right)}}}{C}\right);$$ (4)

$$m_{i,p_{\left(n\right)}}^{\left[t\right]}=m_{i,p_{\left(n\right)}}^{\left[h\right]}\cdot \left(1-\frac{Net_{i,p_{\left(n\right)}}}{C}\right);$$ (5)

- d. Adaptation of the connections $w_{i,j_{\left(n\right)}}$ through the variation $\Delta w_{i,j_{\left(n\right)}}$, which amounts, accordingly, to trapping the energy difference as to Eq (5):

$$\Delta w_{i,j_{\left(n\right)}}=\sum_{p}^{M}\left(m_{i,p_{\left(n\right)}}^{\left[h\right]}-m_{i,p_{\left(n\right)}}^{\left[t\right]}\right)\cdot \left(1-\frac{w_{i,j_{\left(n\right)}}}{C}\right)\cdot m_{j,p_{\left(n\right)}}^{\left[h\right]};$$ (6)

$$w_{i,j_{\left(n+1\right)}}=w_{i,j_{\left(n\right)}}+\alpha \cdot \Delta w_{i,j_{\left(n\right)}}.$$ (7)

First of all, the weights updating will be executed only at every epoch.

Even a cursory comparison of (1) and (5) and (2–3), (6–7), respectively, clearly shows how both steps of the signal transfer process are guided by the same (contraction) principle, and likewise for the two weight adaptation steps (for which we could speak of an energy entrapment principle).

Notice how the term $m_{j,p_{\left(n\right)}}^{\left[h\right]}$ in (6) makes the change in the connection $w_{i,j_{\left(n\right)}}$ proportional to the quantity of free energy by node $m_{i,p_{\left(n\right)}}^{\left[h\right]}$ in favor of node $m_{i,p_{\left(n\right)}}^{\left[t\right]}$. The whole learning process, which essentially consists of a progressive adjustment of the connections aimed at the global minimization of energy, may be seen as a complex juxtaposition of phases of acceleration and deceleration of velocities of the learning signals (adaptations $\Delta w_{i,j_{\left(n\right)}}$ and $\Delta v_{i_{\left(n\right)}}$) inside the ANN connection matrix. To get a clearer understanding of this feature of the AutoCM learning mechanics, begin by considering its convergence condition:

$$\underset{n\to \infty }{\lim}v_{i_{\left(n\right)}}=C$$ (8)

Indeed, when $v_{i_{\left(n\right)}}=C$, then $\Delta v_{i_{\left(n\right)}}=0$ (according to Eq 2), and $m_{j,p_{\left(n\right)}}^{\left[h\right]}=0|\forall p\in M$ (according to Eq 1) and, subsequently, $\Delta w_{i,j_{\left(n\right)}}=0$ (as from Eq 6): the AutoCM then converges.

The matrix w (Eq 7), then, represents the AutoCM knowledge about the whole dataset. Now, if we consider C as a limit value for all the weights of the w matrix, we can write:

$$\begin{array}{l} w_{i,j}^{'}=\frac{w_{i,j}+w_{j,i}}{2};\\ w_{j,i}^{'}=w_{i,j}^{'};\\ d_{i,j}=C-w_{i,j}^{'}\;\text{if}\;i\neq j;\\ d_{i,i}=0. \end{array}$$ (9)

The new matrix *d* is a squared symmetric matrix, where the main diagonal entries are null (i.e., they represent the zero distance of each variable from itself), and where the off-diagonal entries represent ‘distances’ between each couple of variables.

### AutoCM and Minimum Spanning Tree (MST)

Eq (9) transforms the squared weight matrix of AutoCM into a squared matrix of distances among nodes [25]. Each distance between a pair of nodes may therefore be regarded as the weighted edge between these pairs of nodes in a suitable graph-theoretical representation, so that the matrix d itself may be analyzed through the graph theory toolbox.

A graph is a mathematical abstraction that is useful for solving many kinds of problems. Fundamentally, a graph consists of a set of vertices, and a set of edges, where an edge is an object that connects two vertices in the graph. More precisely, a graph is a pair (*V*, *E*), where *V* is a finite set and E is a binary relation on *V*, to which it is possible to associate scalar values (in this case, the distances *d*_*i*,*j*_).

*V* is called a vertex set which elements are called vertices. E is a collection of edges, where an edge is a pair (*u*, *v*) with u, v belonging to V. In a directed graph, edges are ordered pairs, connecting a source vertex to a target vertex. In an undirected graph, edges are un-ordered pairs and connect the two vertices in both directions, hence in an undirected graph (*u*,*v*) and (*v*, *u*) are two ways of writing the same edge.

The graph-theoretical representation is not constrained by any a priori semantic restriction: it does not say what a vertex or edge actually represents. They could be cities with connecting roads, or web-pages with hyperlinks, and so on. These semantic details are irrelevant to determine the graph structure and properties; the only thing that matters is that a specific graph may be taken as a proper representation of the phenomenon under study, to justify attention on that particular mathematical object.

An adjacency-matrix representation of a graph is a 2-dimensional *VxV* array, where rows represent the list of vertices and columns represent edges among vertices. To each element in the array a Boolean value, describing whether the edge (*u*,*v*) is in the graph, is assigned.

A distance matrix among *V* vertices represents an undirected graph, where each vertex is linked with all the others but itself.

At this point, the concept of Minimum Spanning Tree (MST) must be introduced.

The MST problem is defined as follows: find an acyclic subset *T* of *E* that connects all of the vertices in the graph and which total weight (viz., the total distance) is minimized, where the total weight is given by:

$$d(T)=\sum_{i=0}^{N-1}\sum_{j=i+1}^{N}d_{i,j},\forall d_{i,j}$$ (10)

T is called a spanning tree, and the MST is the T whose weighted sum of edges attains the minimum value:

$$Mst=Min\{d(T_{k})\}$$ (11)

Given an undirected graph *G*, representing a matrix of distances *d*, with *V* vertices, completely linked to each other, the total number of their edges (*E*) is:

$$E=\frac{V\cdot (V-1)}{2}$$ (12)

and the number of its possible spanning trees is

$$T=V^{V-2}$$ (13)

Kruskal (1956) found out an algorithm to determinate the MST of any undirected graph in a quadratic number of steps, in the worst case. Obviously, the Kruskal algorithm generates one of the possible MSTs. In fact, in a weighted graph more than one MSTs is possible.

From a conceptual point of view, the MST represents the energy minimization state of a structure. In fact, if we consider the atomic elements of a structure as vertices of a graph and the strength among them as the weight of each edge, linking a pair of vertices, the MST represents the minimum of energy needed so that all the elements of the structure preserve their mutual coherence. In a closed system, all the components tend to minimize the overall energy. So the MST, in specific situations, can represent the most probable state for the system to tend.

To determine the MST of an undirected graph, each edge of the graph must be weighted. Eq (9) shows a way to weight each edge which nodes are the variables of a dataset, and where the weights of a trained AutoCM provide the (weight) metrics.

Obviously, it is possible to use any kind of Auto-Associative ANN or any kind of Linear Auto-Associator to generate a weight matrix among the variables of an assigned dataset. But it is hard to train a two-layer Auto-Associative Back Propagation ANN with the main diagonal weights fixed (to avoid auto-correlation problems). In most cases, the Root Mean Square Error (RMSE) stops to decrease after a few epochs, and especially when the orthogonality of the records is relatively high, a circumstance that is frequent when it is necessary to weight the distance among the records of the assigned dataset. In this case, it is necessary to train the transposed matrix of the dataset. By the way, if a Linear Auto-Associator is used for this purpose, all of the non linear associations among variables would be lost.

Therefore, AutoCM seems to be the best choice to date to compute a complete and a non linear matrix of weights among variables or among records of any assigned dataset.

### AutoCM and the H Function to Measure the Graph Complexity

The Degree of Protection of each node defines the rank of centrality of each node within the graph, when an iterative pruning algorithm is applied. The Pruning Algorithm is a suitable algorithm able to define the degree of protection of each node in any graph [26].

The pruning algorithm can be used also to define the quantity of graph complexity of any graph. If we take *μ* as the mean number of nodes without any link, at each iteration, as the pruning algorithm is running, we can define the hubness Index, *H*_0_, of a graph with *N* nodes. The H Function was described by Buscema et al. at the Semeion Research Center in 2007 [27,28].

In order to properly define this quantity, we need to introduce a few preliminary concepts. A cycle or iteration of the pruning algorithm is defined as a given round of application of the algorithm. At each cycle, corresponds a gradient, which can be different from cycle to cycle. Insofar as two subsequent cycles yield the same gradient, they belong to the same pruning class. As the gradient changes from one cycle to the other, the previous class ends and a new one begins. This allows to define hubness as follows:

$$H_{0}=\frac{\mu \cdot \varphi -1}{A};\;0<H_{0}<2;$$ (14A)

$$\mu =\frac{1}{M}\sum_{i}^{M}Nd_{i}=\frac{N}{M};$$ (14B)

$$\varphi =\frac{1}{P}\sum_{j}^{P}S_{TG_{j}};$$ (14C)

A = number of links of the graph (N-1 for trees);

N = Number of Nodes;

M = number of cycles of the pruning algorithm;

P = number of states implied into a change of gradient, during the pruning process;

Ndi = number of nodes without link at the j-th iteration;

STG j = Summation of the gradient of the states implied into a change of gradient, during the pruning process.

The Eq (14B) measures the mean gradient of the graph.

The Eq (14C) measures the dynamics of the gradient changes during the pruning process.

The Eq (14A) is a complex ratio between the mean gradient and the dynamics of this gradient, from one side, and the global graph connectivity, from the other side.

Using *H*_0_ as a global indicator, it is possible to define to what extent a graph is hub oriented.

Previous studies have shown how the H Function is a suitable algorithm to measure the complexity and the entropy of any a-directed graph [26,27].

### Auto CM and Maximally Regular Graph

The MST represents what we could call the ‘nervous system’ of any dataset. In fact, summing up all of the connection strengths among all the variables, we get the total energy of that system. The MST selects only the connections that minimize this energy, i.e., the only ones that are really necessary to keep the system coherent. Subsequently, all the links included in the MST are fundamental, but, on the contrary, not every ‘fundamental’ link of the dataset needs to be in the MST. Such limit is intrinsic to the nature of MST itself: every link that gives rise to a cycle into the graph (viz., that destroys the graph’s ‘treeness’) is eliminated, whatever its strength and meaningfulness. To fix this shortcoming and to better capture the intrinsic complexity of a dataset, it is necessary to add more links to the MST, according to two criteria: i) the new links have to be relevant from a quantitative point of view; ii) the new links have to be able to generate new cyclic regular microstructures, from a qualitative point of view.

Subsequently, the MST tree-graph is transformed into an undirect graph with cycles. Because of the cycles, the new graph is a dynamic system, involving in its structure the time dimension. This is the reason why this new graph should provide information not only about the structure but also about the functions of the variables of the dataset.

To build the new graph, one needs to proceed as follows: i) assume the MST structure as the starting point of the new graph; ii) consider the sorted list of the connections skipped during the derivation of the MST; iii) estimate the H Function of the new graph each time one adds a new connection to the MST basic structure to monitor the variation of the complexity of the new graph at every step.

The graph which H Function attains the highest value among all the graphs generated by adding back to the original MST, one by one, the missing connections previously skipped during the computation of the MST is defined Maximally Regular Graph (MRG). Starting from Eq (14A), the MRG may be characterized as follows:

$$H_{i}=f(G(A_{i},N))\;/*\;Generic\;Function\;on\;a\;graph\;with\;Ai\;arcs\;and\;N\;nodes\;at\;i-th\;test\;*/$$ (15)

$$H_{i}=\frac{\mu_{i}\cdot \varphi_{i}-1}{A_{i}}\;/*\;Calculation\;of\;H\;Function,\;where\;H\;0\;represents\;MST\;complexity\;*/$$

$$H^{*}=Max\{H_{i}\}\;/*\;Graph\;with\;highest\;H=MRG\;*/$$

$$R^{*}=Max\;\arg\;\{H_{i}\}\;/*\;Number\;of\;links\;added\;by\;MRG\;*/$$

i∈[0,1,2,…,R]/*IndexofHFunction*/

$$N-1<A_{i}<\frac{N\cdot (N-1)}{2}\;/*\;interval\;of\;the\;number\;of\;graph\;arcs\;*/$$

$$R\in \left[0,1,..,\frac{\left(N-1)\cdot (N-2\right)}{2}\right]\;/*\;Number\;of\;the\;skipped\;arcs\;during\;the\;MST\;generation\;*/$$

The R number is a key variable during the computation of the MRG. R could in fact be also null, when the computation of the MST calls for no connections to be skipped. In this case, there is no MRG for that dataset.

R, moreover, makes sure that the last—and subsequently the weakest—connection added to generate the MRG is always more relevant than the weakest connection of the MST. The MRG, finally, generates, starting from the MST, the graph presenting the highest number of regular microstructures that makes use of the most important connections of the dataset. The higher the value of the H Function at the connections selected to generate the MRG, the more meaningful the microstructures of the MRG.

### Activation and Competition System

ACS is an auto-associative neural network, developed by Buscema [28]. ACS is an ANN endowed with an uncommon architecture: any couple of nodes is not linked by a single value, but by a vector of weights, where each vector component comes from a specific metric. Such ‘bio-diversity’ of combinations of metrics can provide interesting results when each metric describes different and consistent details of the same dataset. In this situation, the ACS algorithm forces all the variables to compete among themselves, in different respects.

The ACS algorithm, therefore, is based on the weight matrices of other algorithms. ACS will use these matrices as a complex set of multiple constraints to update its units in response to any input perturbation. ACS, subsequently, works as a dynamic non linear associative memory. Whenever any input is set on, ACS will activate all its units in a dynamic, competitive and cooperative process at the same time. This process will end up when the evolutionary negotiation among all the units will find its natural attractor.

The ACS ANN is a complex kind of Content Addressable Memory (C.A.M.) system. Compared to the classic associative memory by Hinton [29], McClelland and Rumelhart [30] and Grossberg [31–33], ACS presents the following new features: i) The ACS algorithm works using simultaneously many weight matrices, coming from different algorithms and/or ANNs; ii) The ACS algorithm recall is not a one-shot reaction, but an evolutionary process where all its units negotiate their reciprocal value;.

To compute the weight matrices for the ACS algorithm, one can follow different approaches; we will refer to them, respectively, as ‘simple’ and ‘complex’ algorithms. The former entail applying straightforward formulas for association among variables. The latter make use in turn of more ANN architectures to compute weights through a sophisticated learning strategy.

Using ACS we are able to pose some prototypical questions to the assigned dataset, after we trained the whole dataset using the 3 types of algorithms: AutoCM ANN (Eqs 1–9 and see Eq 18), Linear Correlation algorithm (see Eq 16) and Prior Probability algorithm (see Eq 17). ACS, therefore, works using simultaneously 3 different weight matrices.

In detail, we posed two basic questions: i) Which are the prototypical variables connected to the AGA subjects?; ii) Which are the prototypical variables connected to the IUGR subjects?

### ACS Weights: Simple Algorithms

The matrix of associations of M variables from a dataset with N patterns can easily be constructed by computing the linear associations between any couple of the M variables:

$$W_{i,j}^{\left[L\right]}=\frac{\sum_{k=1}^{N}\left(x_{i,k}-{\bar{x}}_{i})\cdot (x_{j,k}-{\bar{x}}_{j}\right)}{\sqrt{\sum_{k=1}^{N}{\left(x_{i,k}-{\bar{x}}_{i}\right)}^{2}\cdot \sum_{k=1}^{N}{\left(x_{j,k}-{\bar{x}}_{j}\right)}^{2}}};$$ (16)

$$-1\leq W_{i,j}^{\left[L\right]}\leq 1;\;i,j\in [1,2,\ldots ,M]$$

The association matrix, $W_{i,j}^{\left[L\right]}$, is a square matrix where all the main diagonal entries are zero. The matrix $W_{i,j}^{\left[L\right]}$ has, however, some limitations. It considers only linear relationships among variables, and it is not sensitive to the frequency and to the distribution of the variables across the dataset. To compensate these limitations, we compute another association matrix, $W_{i,j}^{\left[P\right]}$, based on the distribution probability of co-occurrence of any couple of the M variables:

$$W_{i,j}^{\left[P\right]}=-\ln\frac{\frac{1}{N^{2}}\cdot \sum_{k=1}^{N}x_{i,k}\cdot (1-x_{j,k})\cdot \sum_{k=1}^{N}(1-x_{i,k})\cdot x_{j,k}}{\frac{1}{N^{2}}\cdot \sum_{k=1}^{N}x_{i,k}\cdot x_{j,k}\cdot \sum_{k=1}^{N}\left(1-x_{i,k})\cdot (1-x_{j,k}\right)}$$ (17)

$$-\infty \leq W_{i,j}^{\left[P\right]}\leq +\infty ;\;x\in [0,1];\;i,j\in [1,2,\ldots ,M]$$

If we scale linearly this new matrix, $W_{i,j}^{\left[P\right]}$, in the same interval as for the linear matrix, $W_{i,j}^{\left[L\right]}$, we get two comparable hyper-surfaces into the same metric space.

### ACS Weights: Complex Algorithms

ANNs represent an alternative route, to compute the matrix of the weights connecting the dataset variables. This choice yields two important results. First, we can define each weight taking into account global interactions among variables (i.e., the simultaneous associations among all of them), and not simply coupled interactions as in the association matrices above. Second, we work with nonlinear specifications of the algorithm, that allow to handle even extremely complicated relationships among the dataset variables.

In particular, we considered the Auto-Contractive Maps [22].

Once the AutoCM has been trained, we can transform the trained weight matrix, $w_{i,j_{\left(n\right)}}$, into a new metric as follows:

$$\begin{array}{l} f(x)=\text{the function scales linearly the argument};\;-1\leq x\leq +1;\\ W_{i,j}^{\left[A\right]}=\text{new Auto CM weigths matrix}.\\ W_{i,j}^{\left[A\right]}=f\left(w_{i,j}\right). \end{array}$$ (18)

### Activation & Competition System Algorithm

ACS is a non linear associator, whose cost function is based on the minimization of the energy among units, whenever the system is activated by an external input. Details are below:

$$\begin{array}{l} M=\text{Number of Variables - Units};\\ Q=\text{Number of weights matrices};\\ i,j\in M;\\ k\in Q;\\ W_{i,j}^{k}=\text{value of connection between the i-th and the j-th units of the k-th matrix};\\ Ecc_{i}=\text{global excitation to the i-th unit coming from the other units};\\ Ini_{i}=\text{global inhibition to the i-th unit coming from the other units};\\ E_{i}=\text{final global excitation to the i-th unit};\\ I_{i}=\text{final global inhibition to the i-th unit};\\ {}[n]=\text{cycle of the iteration};\\ u_{i}^{\left[n\right]}=\text{state of the i-th unit at cycle n};\\ H^{\left[n\right]}=\text{amount of units updating at cycle n};\\ Net_{i}=\text{Net Input of the i-th unit};\\ \delta_{i}=\text{delta update of the i-th unit};\\ Input_{i}=\text{value of the i-th external input}:\;-1\leq Input_{i}\leq +1;\\ N_{k,i}^{\left[E\right]}=\text{number of positive weights of the k-th matrix to the i-th unit};\\ N_{k,i}^{\left[I\right]}=\text{number of negative weights of the k-th matrix to the i-th unit};\\ Max=\text{Maximum of activation}:\;Max=1.0;\\ Min=\text{Minimum of activaction}:\;Min=-1.0;\\ Rest=\text{rest value}:\;Rest=-0.1;\\ Decay_{i}^{\left[n\right]}=\text{Decay of activaction the i-th unit at cycle n}\;:Decay_{i}^{\left[n=0\right]}=0.1;\\ \alpha =\text{scalar for the}\;E_{i}\;\text{and}\;I_{i}\;\text{net input to each unit};\\ \beta =\text{scalar for the external input};\\ \varepsilon =\text{a small positive quantity close to zero}. \end{array}$$

$$\begin{array}{l} Ecc_{i}=\alpha \cdot \sum_{k}^{Q}\frac{\sum_{i}^{M}u_{i}^{\left[n\right]}\cdot W_{i,j}^{k}}{N_{k,i}^{\left[E\right]}}\;W_{i,j}^{k}>0;\\ Ini_{i}=\alpha \cdot \sum_{k}^{Q}\frac{\sum_{i}^{M}u_{i}^{\left[n\right]}\cdot W_{i,j}^{k}}{N_{k,i}^{\left[I\right]}}\;W_{i,j}^{k}<0;\\ E_{i}=Ecc_{i}+\beta \cdot Input_{i}\;Input_{i}>0;\\ I_{i}=Ini_{i}+\beta \cdot Input_{i}\;Input_{i}<0;\\ Net_{i}=\left(Max-u_{i}^{\left[n\right]}\right)\cdot E_{i}+\left(u_{i}^{\left[n\right]}-Min\right)\cdot I_{i}-Dec_{i}\cdot \left(u_{i}^{\left[n\right]}-R\;est\right);\\ \delta_{i}=Net_{i}\cdot (1.0-u_{i}^{2});\\ H^{\left[n\right]}=\sum_{i}^{M}\delta_{i}^{2};\\ u_{i}^{\left[n+1\right]}=u_{i}^{\left[n\right]}+\delta_{i};\\ Dec_{i}^{\left[n+1\right]}=Dec_{i}^{\left[n\right]}\cdot e^{-u_{i}^{2}}; \end{array}$$ (19)

*H* ^[*n*]^ is the cost function of ACS to be minimized. Subsequently, when, the algorithm terminates.

More specifically:

$$\left(Max-u_{i}\right)\cdot E_{i}+\left(u_{i}-Min\right)\cdot I_{i}-Dec_{i}\cdot \left(u_{i}-R\;est\right)=0$$

$$Max\cdot E_{i}-u_{i}\cdot E_{i}+u_{i}\cdot I_{i}-Min\cdot I_{i}-Dec_{i}\cdot u_{i}+R\;est\cdot Dec_{i}=0$$

$$\left(-E_{i}+I_{i}-Dec_{i}\right)\cdot u_{i}+Max\cdot E_{i}-Min\cdot I_{i}+R\;est\cdot Dec_{i}=0$$

$$u_{i}=\frac{Max\cdot E_{i}-Min\cdot I_{i}+R\;est\cdot Dec_{i}}{E_{i}-I_{i}+Dec_{i}}$$ (20)

When *Max* = 1; *Min* = −1; *Rest* = 0.1, then:

$$u_{i}=\frac{Ecc_{i}+I_{i}-0.1\cdot Dec_{i}}{Ecc_{i}-I_{i}+Dec_{i}}$$ (21)

We have already said that the ACS ANN is partially inspired to a previous ANN presented by Grossberg [31,33]. But their differences are so marked that we need to present ACS as a new ANN: i) ACS works using simultaneously many weight matrices coming from different algorithms, while Grossberg’ IAC uses only one weight matrix; ii) ACS weight matrices represent different mappings of the same dataset and all the units (variables) are processed in the same way, while Grossberg’ IAC just works when the dataset presents only a specific kind of architecture; iii) The ACS algorithm can use any combination of weight matrices, coming from any kind of algorithm. The only constraint is that all the values of every weight matrix have to be linearly scaled into the same range (typically between -1 and +1), while Grossberg’ IAC can work only with static excitations and inhibitions; iv) Each ACS unit tries to learn its specific value of decay, during its interaction with the other units, while Grossberg’ IAC works with a static decay parameter for all the variables; v) The ACS architecture is a circuit with symmetric weights (vectors of symmetric weights), able to manage a dataset with any kind of variables (Boolean, categorical, continuous, etc.), while Grossberg’ IAC can work only with specific types of variables [31,33].

## Results

### Basic Statistics and Comparisons

The means and the Standard Deviations (SD) of each variable in the subjects investigated are reported in Table 1. No effective difference was found applying a T-Student’s test, thus, the two samples were quite similar (Tau = 1.7867 and p = 0.050770 for the means and Tau = 1.7377 and p = 0.055069 for the SDs). The matrix of linear correlation among variables is shown in Table 2. From this table we derived a T-Test of the comparison between R Squared of each variable in the IUGR and appropriate for gestational age (AGA) samples, respectively, which is reported in Table 3. For all variables the difference between the two subgroups is not statistically significant with the exception of mRNA_IL6 (p = 0.0386), mRNA_IGF1 (p = 0.0386), and PLAIL6 (p = 0.0537).

**Table 1:** Basic statistics: means and SDs of each single variable, Males (M) and females (F) in the two classes, intra-uterine growth retardation (IUGR) and appropriate for gestational age (AGA).

Variable	Mean IUGR	Mean AGA	SD IUGR	SD AGA
**Gest Age (wk)**	33,26	36,75	3,49	2,57
**PRO (**μ**g/mg)**	35,39	33,064	22,69	21,56
**mRNA_BP1**	0,069	0,00	0,22	0,00
**mRNA_BP2**	0,10	0,02	0,22	0,01
**mRNA_IL6**	0,25	0,09	0,26	0,07
**mRNA_IGF1**	0,25	0,09	0,26	0,07
**mRNA_IGF2**	0,16	0,12	0,22	0,08
**PLA_IGF2 (ng/mg)**	163,83	134,16	31,46	27,12
**PLATNF (ng/mg)**	1,94	3,36	1,77	2,06
**PLAIL6 (ng/mg)**	62,20	44,38	34,11	21,53
**PLA_BP2 (ng/mg)**	110,40	83,44	96,36	73,38
**SEX**	11M/ 9F	12M/14F		

IUGR: intra-uterine growth retardation; AGA: appropriate for gestational age; Gest Age: gestational age; PRO: total protein content per mg of placental tissue; mRNA_BP1: IGF Binding Protein-1 relative gene expression; mRNA_BP2: IGF Binding Protein-2 relative gene expression; mRNA_IL6: Interleukin-6 relative gene expression; mRNA_IGF1: Insulin-like growth factor-1 relative gene expression; mRNA_IGF2: Insulin-like growth factor-2 relative gene expression; PLA_IGF2: Insulin-like growth factor-2 normalized placental lysate concentration; PLATNF: Tumor Necrosis Factor-α normalized placental lysate concentration; PLAIL6: Interleukin-6 normalized placental lysate concentration; PLA_BP2: IGF Binding Protein-2 normalized placental lysate concentration; males (M) and females (F).

**Table 2:** Matrix of linear correlation among the variables in the study.

**IUGR**	-0.04	0.04	-0.51	0.05	0.23	0.29	0.41	0.41	0.15	0.46	-0.35	0.31	0.16	-1.00	1.00
**AGA**	0.04	-0.04	0.51	-0.05	-0.23	-0.29	-0.41	-0.41	-0.15	-0.46	0.35	-0.31	-0.16	1.00	-1.00
**PLA_BP2**	0.19	-0.19	-0.06	0.18	-0.14	-0.20	-0.21	-0.21	-0.20	0.36	-0.11	0.10	1.00	-0.16	0.16
**PLAIL6**	-0.04	0.04	-0.19	-0.03	-0.15	-0.11	0.43	0.43	-0.14	0.37	0.04	1.00	0.10	-0.31	0.31
**PLA TNF**	0.13	-0.13	0.43	0.03	-0.14	-0.17	-0.19	-0.19	-0.01	-0.18	1.00	0.04	-0.11	0.35	-0.35
**PLA _IGF2**	0.15	-0.15	-0.14	0.02	0.03	0.00	0.16	0.16	0.01	1.00	-0.18	0.37	0.36	-0.46	0.46
**mRNA_IGF2**	-0.04	0.04	-0.30	-0.29	-0.03	0.00	-0.11	-0.11	1.00	0.01	-0.01	-0.14	-0.20	-0.15	0.15
**mRNA_IGF1**	0.03	-0.03	-0.17	0.24	0.32	0.44	1.00	1.00	-0.11	0.16	-0.19	0.43	-0.21	-0.41	0.41
**mRNA_IL6**	0.03	-0.03	-0.17	0.24	0.32	0.44	1.00	1.00	-0.11	0.16	-0.19	0.43	-0.21	-0.41	0.41
**mRNA_BP2**	0.12	-0.12	-0.01	0.46	0.96	1.00	0.44	0.44	0.00	0.00	-0.17	-0.11	-0.20	-0.29	0.29
**mRNA_BP1**	0.12	-0.12	0.01	0.43	1.00	0.96	0.32	0.32	-0.03	0.03	-0.14	-0.15	-0.14	-0.23	0.23
**PRO**	0.15	-0.15	0.20	1.00	0.43	0.46	0.24	0.24	-0.29	0.02	0.03	-0.03	0.18	-0.05	0.05
**Gest Age**	0.00	0.00	1.00	0.20	0.01	-0.01	-0.17	-0.17	-0.30	-0.14	0.13	-0.19	-0.06	0.51	-0.51
**Female**	-1.00	1.00	0.00	-0.15	-0.12	-0.12	-0.03	-0.03	0.04	-0.15	-0.13	0.04	-0.19	-0.04	0.04
**Male**	1.00	-1.00	0.00	0.15	0.12	0.12	0.03	0.03	-0.04	0.15	0.13	-0.04	0.19	0.04	-0.04
**Correlation all**	**Male**	**Female**	**GestAge**	**PRO micro/mg**	**mRNA_BP1**	**mRNA_BP2**	**mRNA_IL6**	**mRNA_IGF1**	**mRNA_IGF2**	**PLA_IGF2**	**PLA TNF**	**PLA IL6**	**PLA_BP2**	**AGA**	**IUGR**

IUGR: intra-uterine growth retardation; AGA: appropriate for gestational age; Gest Age: gestational age (week); PRO: total protein content per mg of placental tissue (μg/mg); mRNA_BP1: IGF Binding Protein-1 relative gene expression; mRNA_BP2: IGF Binding Protein-2 relative gene expression; mRNA_IL6: Interleukin-6 relative gene expression; mRNA_IGF1: Insulin-like growth factor-1 relative gene expression; mRNA_IGF2: Insulin-like growth factor-2 relative gene expression; PLA_IGF2: Insulin-like growth factor-2 normalized placental lysate concentration (ng/mg); PLATNF: Tumor Necrosis Factor-α normalized placental lysate concentration (ng/mg); PLAIL6: Interleukin-6 normalized placental lysate concentration (ng/mg); PLA_BP2: IGF Binding Protein-2 normalized placental lysate concentration (ng/mg).

**Table 3:** T-Test between R Squared of the variables analyzed in intra-uterine growth retarded (IUGR) and appropriate for gestational age (AGA) newborns.

*Variable*	R^2 between IUGR and AGA	Test-T (p)
GEST AGE	0.26	0.3707
PRO	0.00	0.3422
mRNA_BP1	0.05	0.1384
mRNA_BP2	0.08	0.1882
mRNA_IL6	0.17	0.0386*
mRNA_IGFI	0.17	0.0386*
mRNA_IGF2	0.02	0.3383
PLA_IGF2	0.21	0.1066
PLATNF	0.12	0.1485
PLAIL6	0.10	0.0537*
PLA_BP2	0.03	0.337

Gest Age: gestational age (week); PRO: total protein content per mg of placental tissue (μg/mg); mRNA_BP1: IGF Binding Protein-1 relative gene expression; mRNA_BP2: IGF Binding Protein-2 relative gene expression; mRNA_IL6: Interleukin-6 relative gene expression; mRNA_IGF1: Insulin-like growth factor-1 relative gene expression; mRNA_IGF2: Insulin-like growth factor-2 relative gene expression; PLA_IGF2: Insulin-like growth factor-2 normalized placental lysate concentration (ng/mg); PLATNF: Tumor Necrosis Factor-α normalized placental lysate concentration (ng/mg); PLAIL6: Interleukin-6 normalized placental lysate concentration (ng/mg); PLA_BP2: IGF Binding Protein-2 normalized placental lysate concentration (ng/mg).

*p<0.05.

The best of 1000 K-Means Clustering is shown in Table 4. Despite the fact that the clustering validity indexes were good, the K-mean did not adequately separate AGA and IUGR subjects, with sensitivity and specificity performances ranging between 46% and 54%.

**Table 4:** K-Mean Clustering of intra-uterine growth retarded (IUGR) and appropriate for gestational age (AGA) subjects.

Silhouette Index:	0.469548	-1<S(i)<1	S(Best) = +1
Davies-Bouldin Index:	0.938545	0<DB(i)<+inf	DB(Best = 0)
**Cluster #1**			
AGA 3	IUGR 1		
AGA 5	IUGR 2		
AGA 6	IUGR 6		
AGA 7	IUGR 9		
AGA 8	IUGR 12		
AGA 9	IUGR 13		
AGA 13	IUGR 14		
AGA 18	IUGR 16		
AGA 20	IUGR 17		
AGA 23	IUGR 18		
AGA 24	AGA 25		
**46% AGA—50% IUGR**			
**Cluster #2**			
AGA 1	IUGR 3		
AGA 2	IUGR 4		
AGA 4	IUGR 5		
AGA 10	IUGR 7		
AGA 11	IUGR 8		
AGA 12	IUGR 10		
AGA 14	IUGR 11		
AGA 15	IUGR 15		
AGA 16	IUGR 19		
AGA 17	IUGR 20		
AGA 19	AGA22		
AGA 21	AGA 26		
**54% AGA—50% IUGR**			

The subsequent results of the classification between the two diagnostic classes, obtained by Linear Discriminant Analysis (LDA) applied to Principal Component Analysis (PCA) weighed values as input vectors, are shown as Confusion Matrix (Table 5).

**Table 5:** Confusion Matrix of the results obtained to classify intra-uterine growth retarded (IUGR) and appropriate for gestational age (AGA) newborns using linear discriminant analysis (LDA) applied to principal component analysis (PCA).

Conf Mat	AGA	IUGR	Total	Errors	Correct classification
**AGA**	18	8	26	8	69.23%
**IUGR**	12	8	20	12	40.00%
**Total**	30	16	46	20	
**Aritmetic Mean Accuracy**	54.62%				
**Weighted Mean Accuracy**	56.52%				

These preliminary analyses supported the need for a more complex analysis to discriminate and understand further information embedded in the dataset.

### Application of Auto-Contractive Map to the Dataset

**Fig 1:**
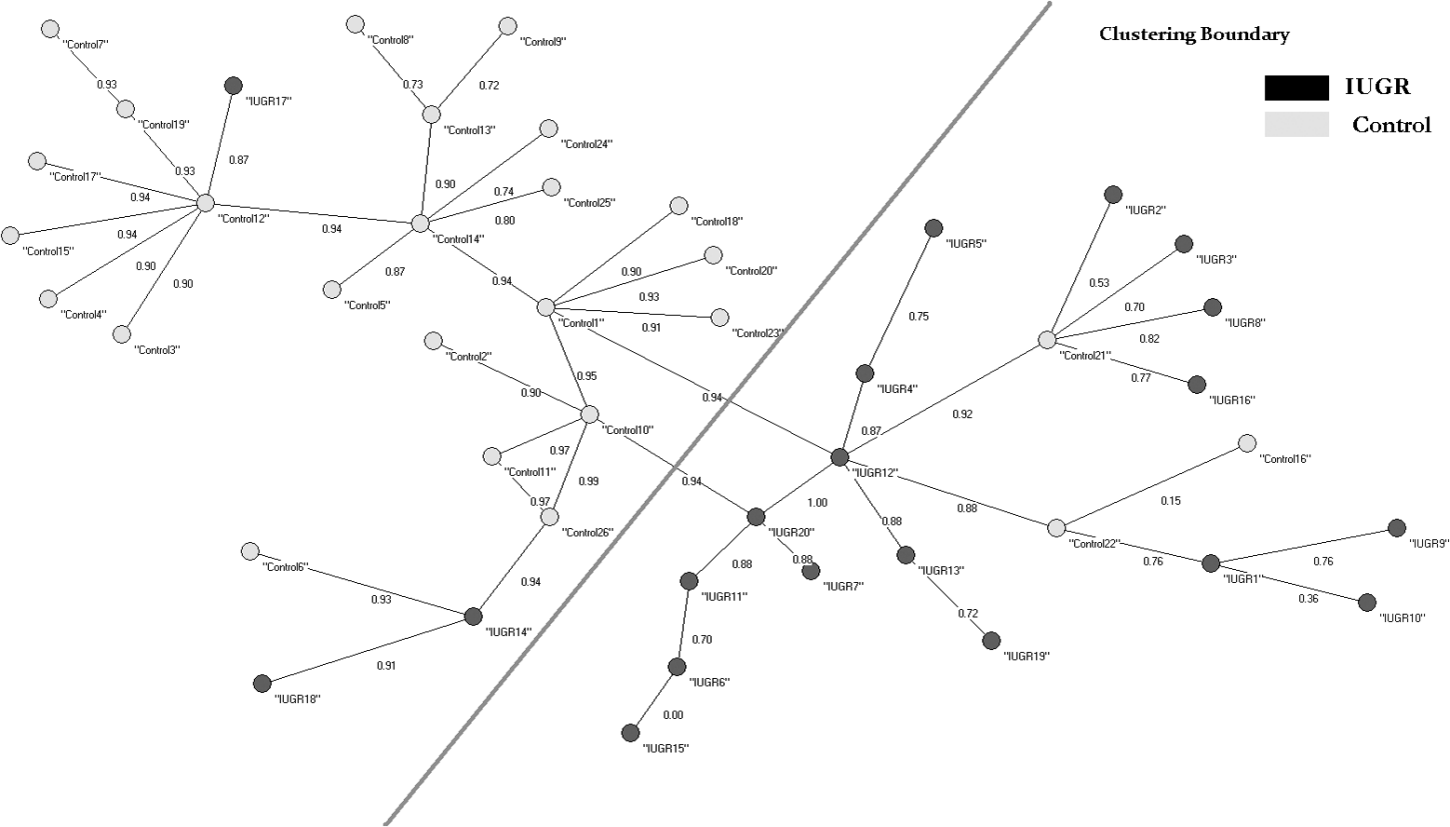
Auto CM and MRG Blind Test on the Records (Red = AGA–Blue = IUGR). The accuracy in identifying IUGR was 85%, and in identifying AGA 88.46%.

First, auto-contractive Map (AutoCM) Artificial Neural Network, was used to cluster the records in a blind test. This clustering was effective (Fig 1), and was used to understand the meaning of each variable in the dataset: 88.46% of AGA and 85% of IUGR were clustered correctly. Subsequently, AutoCM was able to find important features in the dataset, and to distinguish the two samples by using only the 12 independent variables. These features were invisible to traditional algorithms.

Although the clustering validity indexes were good, the K-mean confused, however, AGA and IUGR subjects, with sensitivity and specificity performances ranging between 46% and 54%. The emerging confusion matrix derived from this classification task is shown in Table 6.

**Table 6:** Confusion matrix of Auto-CM clustering shown in Fig 1.

Conf Mat	AGA	IUGR	Total	Errors	Correct classification
**AGA**	23	3	26	3	88.46%
**IUGR**	3	17	20	3	85.00%
**Total**	26	20	46	6	
**Aritmetic Mean Accuracy**	86.73%				
**Weighted Mean Accuracy**	86.96%				

IUGR: intra-uterine growth retardation; AGA: appropriate gestational for age newborns.

**Fig 2:**
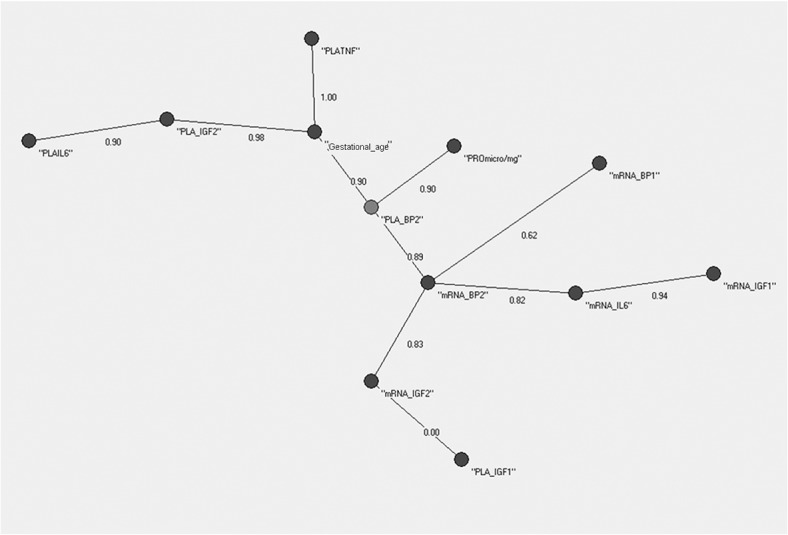
AutoCM applied to the 26 AGA newborns. From a medical point of view this suggests that the placental IGFBP-2 content is a key point related with normal fetal growth.

**Fig 3:**
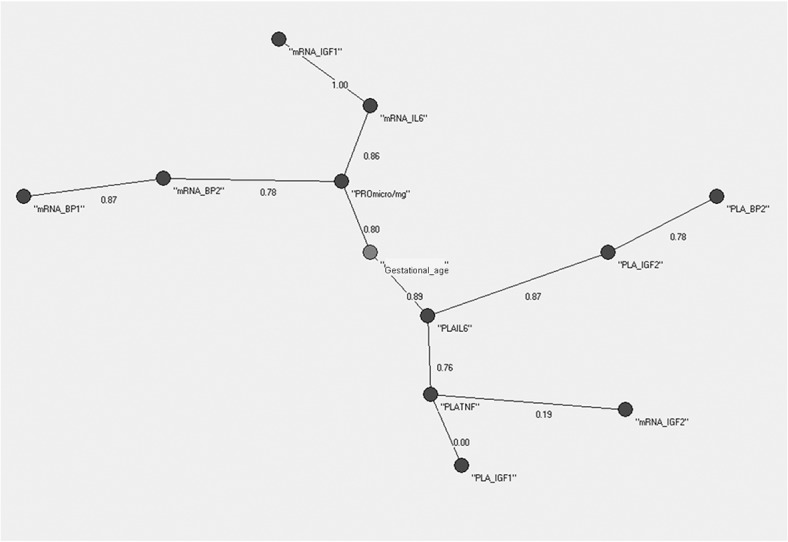
AutoCM applied to the 20 IUGR newborns. This tree suggests “gestational age” as a key point in intra-uterine growth retardation. This, however, is related to the fact that many IUGR subjects are often born premature, and does not provide a biological explanation yet for abnormal fetal growth.

In an independent way, the Minimum Spanning Trees (MSTs) of AutoCM was then applied to the 12 variables of the dataset of AGA and IUGR, and results are shown in Figs 2 and 3 with minor differences emerging. In detail, the center of the tree in the AGA MST (Fig 2) was the variable “PLA_BP2” (IGFBP-2 placental content per mg of placental tissue), while the center for the IUGR MST was the variable “Gestational Age” (Fig 3). In the AGA MST (Fig 2) the variable “PLA_BP2” was connected to the variables “mRNA_BP2” (IGFBP-2 relative gene expression), “Gestational Age”, PRO μg/mg” (total protein content per mg of placental tissue) while in the IUGR MST (Fig 3) the same variable was a lateral leaf, connected to the variable “PLA_IGF2” (IGF2 placental content per mg of placental tissue).

**Fig 4:**
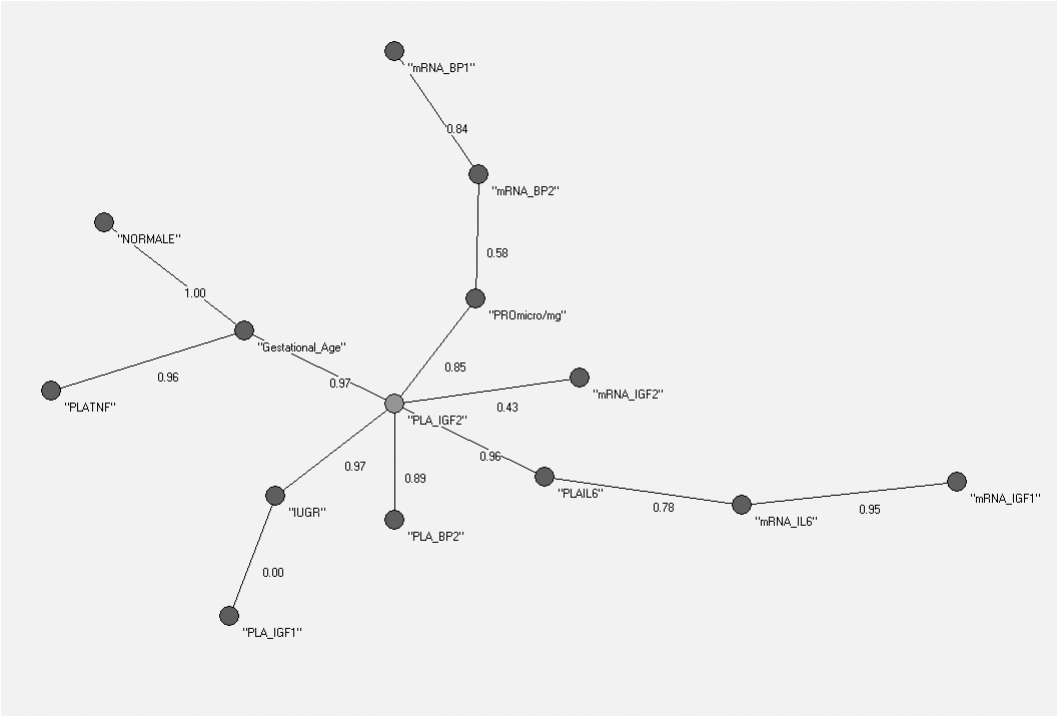
AutoCM applied to the global dataset (AGA plus IUGR). This represents IGF-2 a the key peptide for fetal growth in all conditions.

The MST of the AutoCM algorithm applied to the entire dataset (14 variables) is shown in Fig 4. “PLA_IGF2” (IGF2 placental content per mg of placental tissue) became the central variable in this representation.

However, the AutoCM did not discriminate sufficiently the two samples, and thus, we used a more powerful algorithm to enhance the dynamics of the AutoCM weight matrix.

### Activation and Competition System Applied to the Dataset

Using Activation and Competition System (ACS) we were able to put some prototypical questions in the assigned dataset, after we trained the whole dataset using the 3 types of algorithms: AutoCM ANN (Eqs 1–9 and Eq 18), Linear Correlation algorithm (Eq 16) and Prior Probability algorithm (Eq 17) (For equations see materials and methods).

**Fig 5:**
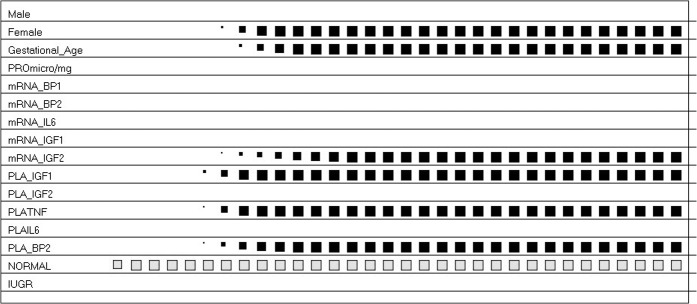
The Activation and Competition System (ACS) and AGA. ACS defined the profile below for the AGA subjects (read from left to right). In brief, the condition of AGA, i.e. normal fetal growth and pregnancy, was described by the amount of IGF-2 relative gene expression, and by IGFBP-2 and TNF-α placental contents.

**Fig 6:**
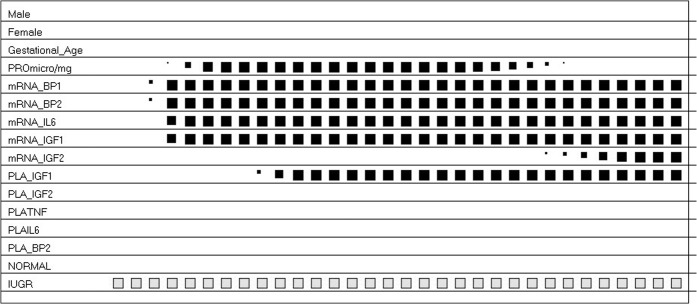
The Activation and Competition System (ACS) And IUGR. ACS defined the profile below for the IUGR subjects (read from left to right). In brief, the condition of IUGR, i.e. intra-uterine growth retardation, was characterized by changes in IGF-I, IGFBP-1, IGFBP-2 and IL-6 gene expression in placenta, with a minor role for total protein content.

The dynamics of ACS to design the profile of the prototypical AGA subjects and IUGR subjects are shown in Figs 5 and 6, respectively. Table 7 reports the final prototypes.

**Table 7:** Final Prototype of appropriate for gestational age (AGA) and intrauterine growth retarted (IUGR) subjects using the activation and competition system (ACS).

*Variables*	AGA (Cycles 1732)	IUGR (Cycles 4818)
**Male**	-0.89	-1.00
**Female**	1.00	-0.93
**Gest Age**	1.00	-1.00
**PRO**	-1.00	-0.37
**mRNA_BP1:**	-1.00	1.00
**mRNA_BP2:**	-1.00	1.00
**mRNA_IL6:**	-1.00	1.00
**mRNA_IGFI:**	-1.00	1.00
**mRNA_IGF2:**	1.00	1.00
**PLA_IGF2:**	-0.98	-1.00
**PLATNF:**	1.00	-1.00
**PLAIL6:**	-1.00	-0.83
**PLA_BP2:**	1.00	-1.00

The numbers refer to the fuzzy membership belonging to the AGA and IUGR class from -1 (minimum membership) to 1 (maximum membership).

Gest Age: gestational age (week); PRO: total protein content per mg of placental tissue (μg/mg); mRNA_BP1: IGF Binding Protein-1 relative gene expression; mRNA_BP2: IGF Binding Protein-2 relative gene expression; mRNA_IL6: Interleukin-6 relative gene expression; mRNA_IGF1: Insulin-like growth factor-1 relative gene expression; mRNA_IGF2: Insulin-like growth factor-2 relative gene expression; PLA_IGF2: Insulin-like growth factor-2 normalized placental lysate concentration (ng/mg); PLATNF: Tumor Necrosis Factor-α nor.malized placental lysate concentration (ng/mg); PLAIL6: Interleukin-6 normalized placental lysate concentration (ng/mg); PLA_BP2: IGF Binding Protein-2 normalized placental lysate concentration (ng/mg).

The emerging picture was that IL-6, Tumor necrosis factor (TNF)-α, and IGF system peptides in placenta, although with some differences, were important factors in intra-uterine growth, both in conditions of appropriate fetal growth and intra-uterine growth restriction.

## Discussion

The first basic idea of this study was simple: to identify as much as possible of the key information biologically grounded in this dataset which was still hidden. The linear algorithms used commonly in the literature consider only the blatant information and the key information is considered “noise”. We supported the idea that the AutoCM algorithm was able to understand which part of the so called noise was key information, providing the fundamental associations among variables and records (patients or cases).

The second idea of this study was to demonstrate that a dataset is only a static snapshot of a specific situation; using ACS algorithm we showed how further hidden information could actually emerge by means of dynamic and non-linear interactions among variables, constrained by suitable parameters. The basic idea was to transform a dataset, using suitable non-linear algorithms, as ACS, into a simulation environment to test hypotheses, considering how each variable could negotiate its value dynamically with the others. In other words, any dataset becomes a virtual content addressable memory.

This study re-explored the associations between IGF system peptides and their correspondent relative gene expression, and two pro-inflammatory cytokines, namely IL-6 and TNF-α, in placenta in relationship with appropriate and restricted fetal growth using complementary non-linear approaches: a semantic connectivity map and a prototypical discriminating variable profile.

The highlights of this study with regard to the mathematical approach were represented by two main findings: a) semantic connectivity maps, usually devoted to variable mapping, could be successfully applied to records in the attempt to cluster and differentiate different conditions under study (in this case normal fetal growth and fetal growth retardation); b) the interrogation of the study variables with non-linear associative memory algorithms allowed to develop variable profiles which discriminated the two conditions under study better than any other form of analysis based on classical statistics (K means) or even artificial adaptive systems as Auto-CM.

From a medical and biological point of view this study showed, among the variables studied, that the condition of AGA, i.e. normal fetal growth and pregnancy, was explained by IGF-2 relative gene expression, and by IGFBP-2 and TNF-α placental contents. IUGR instead was explained by IGF-I, IGFBP-1, IGFBP-2 and IL-6 gene expression in placenta, with a minor role for total protein content.

Therefore, at variance with our previous analyses we could finally establish that TNF-α was implicated in normal fetal growth in addition to IGF-2 and IGFBP-2, whereas in IUGR, IL-6 was the implicated cytokine in combination with IGF-I, IGFBP-1 and IGFBP-2. Interestingly, previous analyses [4,16] did not identify any clear role for IGFBP-1.

AGA was explained by IGF-2, as expected, and by IGFBP-2. In vitro, animal, and human studies have repeatedly showed that IGF-2 was an important determinant of fetal growth [13–16].

IGFBP-2 is known to have an inhibitory action on IGFs, however, in recent years independent effects on glucose metabolism have been shown also [34], and in obesity, for example, it has been shown to reflect long-term insulin sensitivity [35]. Therefore, IGFBP-2 could have yet unknown effects in utero on fetal growth and on placental metabolism. Altogether, to date, IGFBP-2 has been poorly studied, and previously has not been considered an important bio-regulator of IGF bio-availability [12]. In cord serum, we previously showed a positive relationship of IGF-2, and negative relationship of IGFBP-2 on both birth length and weight [36].

As to TNF-α, data in the literature are contrasting. Some studies reported unchanged TNF-α mRNA expression in human placenta in IUGR compared with controls [37] whereas others reported increased TNF-α in the perfusate of IUGR placentas [37]. TNF-α was reported to be increased in the serum and in the amniotic fluid of mothers with fetuses suffering of IUGR [38,39]. Our data clearly suggested an important effect on normal fetal growth. Interestingly, recent in vitro data, in throphoblast cells, showed that TNF-α was able to induce a loss of sensitivity to IGF-I stimulation [39], and we observed a key-role for IGF-I in IUGR but not in AGA where TNF-α seemed to be so relevant.

In IUGR the key-players in placenta resulted largely different. An effect of IGF-I was shown that was not evident in AGA, and besides IGFBP-2 an effect of IGFBP-1 was also evidenced. This latter finding was in agreement with published experimental data [40,41].

IL-6 has been studied only recently and few data are available [13]. This study confirmed a central role of IL-6 content in placenta in IUGR [4,13,16]. We showed previously that IL-6 mRNA was significantly increased in the placenta of IUGR neonates [13]. This pro-inflammatory cytokine was of particular interest as interactions with the IGF system in many chronic inflammatory diseases have been reported [41–44], and interesting molecular mechanisms of insulin-resistance shown [45–49]. Insulin-resistance is considered to be the cause of the metabolic syndrome in later life, and subjects born IUGR have been shown to have a greater prevalence of this condition compared with subjects born AGA.

In summary, these analyses showed that IL-6, TNF-α, and IGF system peptides in placenta, although with some differences, were important factors in intra-uterine growth, both in conditions of appropriate and restricted fetal growth. The data overall offered a further insight into placental players of fetal growth within the IGF and cytokine systems, and provided new information with respect to our previous analyses. Moreover, this kind of data could provide useful information for directions of future research and potential therapeutic targets.

The Validity of AutoCM has been addressed in a number of papers published in other biological fields [21–23], and the method has been bench-marked in previous publications against supervised and unsupervised machine learning [50, 51]. The only example available in addition to that described in this paper is related to an experience carried out in radiotherapy applied to children with brain cancer [28].

The specificity of the example provided with intrauterine growth retardation is linked to the application of algorithms to the variables and to the records.

Finally, we think the following conclusions could be drawn also: i) the AutoCM Algorithm in comparison with the other algorithms known in the literature, both linear and non-linear, is able to cluster in the best way IUGR and AGA subjects using the biological input of a dataset; ii) the AutoCM Algorithm provides simultaneously the networks of similarities within a dataset, in which medical doctors can see: a) the similarities of each newborn with the others; b) which newborns are the prototypes of the others (the Hubs); c) which newborns are in the grey zone of disease and which are clearly IUGR or clearly AGA (position of the newborn in the networks); iii) the AutoCM Algorithm provides also the networks of the variables describing the dataset. iv) ACS Algorithm, further, would allow specialists to put semantic queries into a dataset, to discover the prototypical features of each variable in the dataset, when one or more variables are activated dynamically. ACS, then, could transform the dataset into a dynamical system for a data driven simulation environment, selecting the vulnerable and the protective variables determining IUGR.

## Supporting Information

S1 Dataset Supplementary table of the data for all 14 variables for all 46 patients included in the analyses of this study. (DOC)
