# Supplementary figures and images for: Data Mining of Determinants of Intrauterine Growth Retardation Revisited Using Novel Algorithms Generating Semantic Maps and Prototypical Discriminating Variable Profiles
Source: PLoS One. 2015 Jul 9;10(7):e0126020. doi: 10.1371/journal.pone.0126020 (PMC4497659; doi:10.1371/journal.pone.0126020)

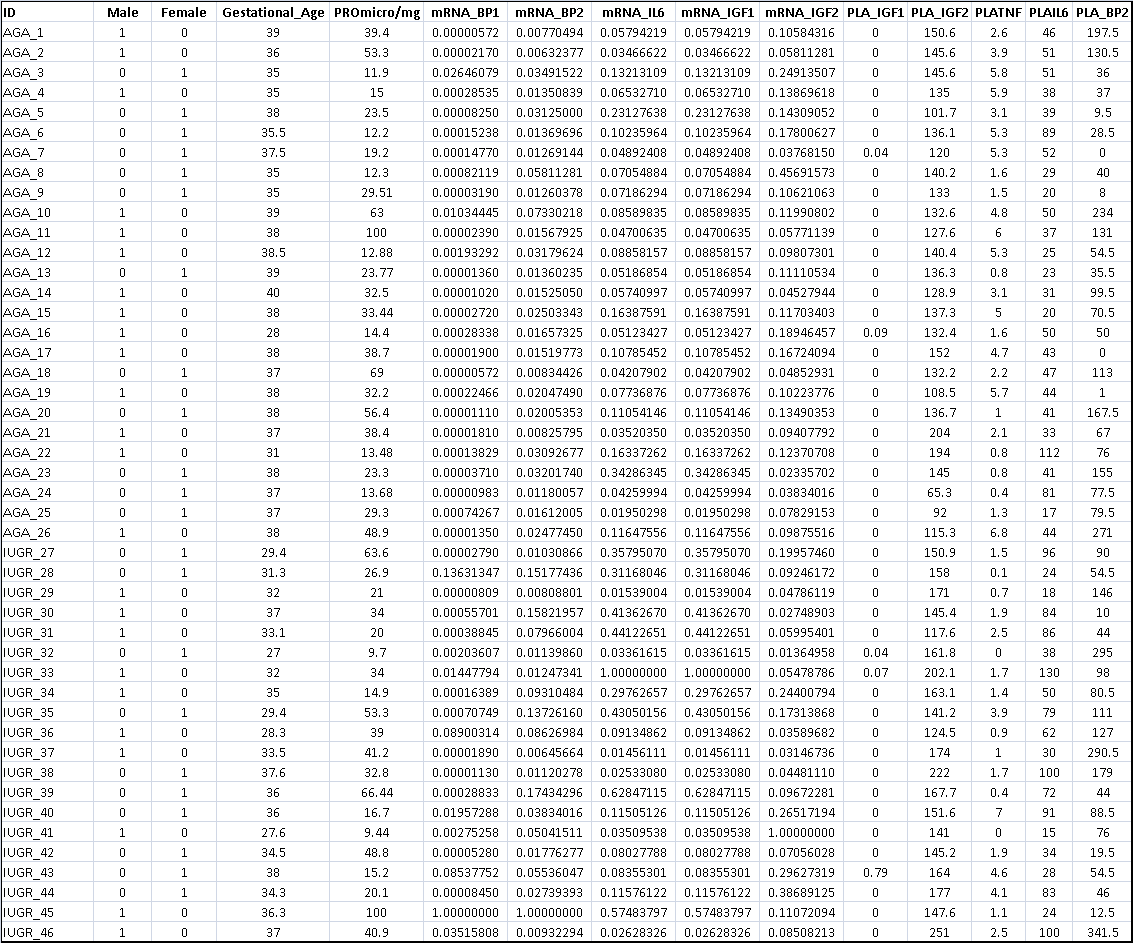

Supplement: S1 Dataset — (DOC) [file pone.0126020.s001.doc]
